# Aquatic therapy in congenital malformation during the use of external fixator for bone lengthening: It is possible?

**DOI:** 10.1016/j.clinsp.2024.100416

**Published:** 2024-06-18

**Authors:** Caio Roberto Aparecido de Paschoal Castro, Alessandra Mitie Kakihata, Carla Borges Fleuri de Barros, Monica Gonçalves, Beatriz Carvalho de Souza, Larissa Soares da Silva, Douglas Martins Braga

**Affiliations:** Setor de Fisioterapia Aquática da Associação de Assistência à Criança Deficiente (AACD), São Paulo, SP, Brasil

**Keywords:** Congenital Anomalies, Bone Lengthening, Aquatic Therapy

## Abstract

**Objectives:**

The objective of this study is to describe the rehabilitation of individuals with Congenital Malformations (CMF) during the use of an External Fixator (EF) in Aquatic Therapy (AT) and to analyze the association between diagnosis, EF type and location with rehabilitation process outcomes, surgical intervention, and adverse effects.

**Methods:**

This retrospective study included 29 medical records from which the personal and rehabilitation data of the patient were collected. The AT used was described and the outcome variables were associated. The medical records were selected by screening the database of the CMF clinic at the AACD. The inclusion criteria were participants with CMF who used EF treated between 2011 and 2019 of both genders and without age restriction. The exclusion criteria were incomplete medical record data or not undergoing AT while using EF. The extracted data included diagnosis, gender, age, EF type and location, objective of the surgery, adverse events, surgical interventions, time of rehabilitation in AT, physiotherapeutic objectives, and rehabilitation process outcomes in AT.

**Results:**

The mean age of the participants was 12.1 ± 3.99 years, with male predominance (55 %) and hemimelia cases (37 %). The most used EF was circular (51 %), located in the femur (37 %), and the main objective of surgery was bone lengthening (52 %). The most recurrent adverse effect was infection (62 %) and 76 % completed AT. There was no association between the variables analyzed.

**Conclusions:**

It was possible to describe CMF rehabilitation with EF in AT. There was no association between the variables analyzed.

## Highlights

• Description of the rehabilitation of individuals with congenital malformations, with external fixator in aquatic therapy.
• There is no association between diagnosis, type, and location of the external fixator, with the outcomes evaluated.
• Aquatic therapy is a safe treatment option for the population studied.

## Introduction

Congenital Malformations (CMF) are functional or structural anomalies resulting from errors in human morphogenesis that can change the musculoskeletal system.^1^ Some of these changes shorten the limb, as in some syndromes, congenital deficiencies, and hemimelia cases. In these conditions, the use of External Fixators (EF) is recommended for bone lengthening and/or deformity corrections.^2^

EF has been used to treat osteoarticular disorders since 1853.^3^ Circular and unilateral EF are the most used in people with CMF. Circular EF helps form not only bone tissue but also soft tissue,^4^ and unilateral EF is more suitable for simple lengthening as it often does not support excessive lengthening, presenting secondary misalignment and premature consolidation as potential complications.^5^

Changes such as pain, decreased Range of Motion (ROM), pin-site infections, and fractures are expected during the use of the EF. Bone and soft tissue complications may occur, compromising functionality. The severity of these complications varies with diagnosis, the extent of lengthening or correction, tissue quality, surgical technique, and EF used. Due to these changes, multidisciplinary rehabilitation is essential for the success of this treatment.^2^

Of the therapeutic modalities, Aquatic Therapy (AT) has specific benefits because of the distinguished hydrodynamic properties and physical principles of the liquid environment, which optimizes gains and improves the desired results.^5^ This environment allows for a differentiated approach, increasing freedom of movement and facilitating muscle activation.^6^ The effects of water on the immersed body can improve or hinder the activities proposed by the physiotherapist.

Warm water between 32–35°C has an analgesic effect, causing vasodilation, improving systemic blood circulation, and resulting in greater nutrient supply to the tissues. In addition, it improves the ROM due to muscle relaxation. Buoyancy is a force that acts as a vector against the action of gravity and is directly proportional to the individual's level of immersion, decreasing body weight.^6^ Body density is another factor that influences buoyancy as the denser the body, the lower its ability to float. Hydrostatic pressure assists in venous return, increasing its action with depth. Viscosity is the magnitude of friction between the body and the fluid, which increases as the body increases its speed of motion. The response time to imbalances increases during immersion, providing and helping the participant with challenging activities.^7^^,^^8^

The literature shows few studies on the use of AT for people with CMF using EF. Only one article was found that describes the prevalence of infection in individuals with an external fixator in the pool, but not only in individuals with CMF.^9^ Therefore, the objectives of this study are to describe the rehabilitation process of participants with CMF during the use of EF in AT at the Associação de Assistência à Criança Deficiente (AACD) from 2011 to 2019, and to analyze if there are associations between EF type and location and diagnosis with new surgical interventions, expected adverse effects (pain, fracture, infection, and limited ROM), and rehabilitation process outcomes in AT.

## Material and methods

This retrospective observational study was approved by the AACD Research Ethics Committee (CAAE: 34269420.7.0000.0085). The authors signed a confidentiality agreement, and the data were collected so as not to identify the research participants. The study followed the recommendations of Strengthening the Reporting of Observational Studies in Epidemiology (STROBE).

The medical records were selected by screening the database of the CMF clinic at the AACD. The inclusion criteria were participants with CMF who used EF treated at the AACD between 2011 and 2019 of both genders and without age restriction. The exclusion criteria were incomplete medical record data or not undergoing AT while using EF.

The extracted data included diagnosis, gender, age, EF type and location, objective of the surgery, adverse events, surgical interventions, time of rehabilitation in AT, physiotherapeutic objectives, and rehabilitation process outcomes in AT.

The time the participant performed AT before placing or after removing the EF was disregarded. If the participant performed AT without interruptions, the outcome was considered reached and, if the participant was removed from AT due to absences, clinical complications, or the need for a surgical procedure, the outcome was not considered achieved.

### Data analysis

The Shapiro-Wilk test was used to analyze the Gaussian distribution of the sample characterization numerical data (age, time with EF, time in AT). These data were presented as mean and standard deviation, considering a confidence interval of 95 %. Categorical sample characterization data were expressed as a percentage (gender, diagnosis, EF type and location, and objective of the surgery). Data related to physiotherapeutic objectives were expressed as percentages. The Pearson's Chi-Square test was used to analyze the association between categorical variables with a 2×2 contingency table, and the Row × Column test when the contingency table had more than two variables in the row or column. A confidence interval of 95 % and p < 0.05 were considered. All data were collected and stored in an Excel Office 2010 spreadsheet. The SPSS software 28.0.0 was used for the statistical analysis.

## Results

**Fig. 1:**
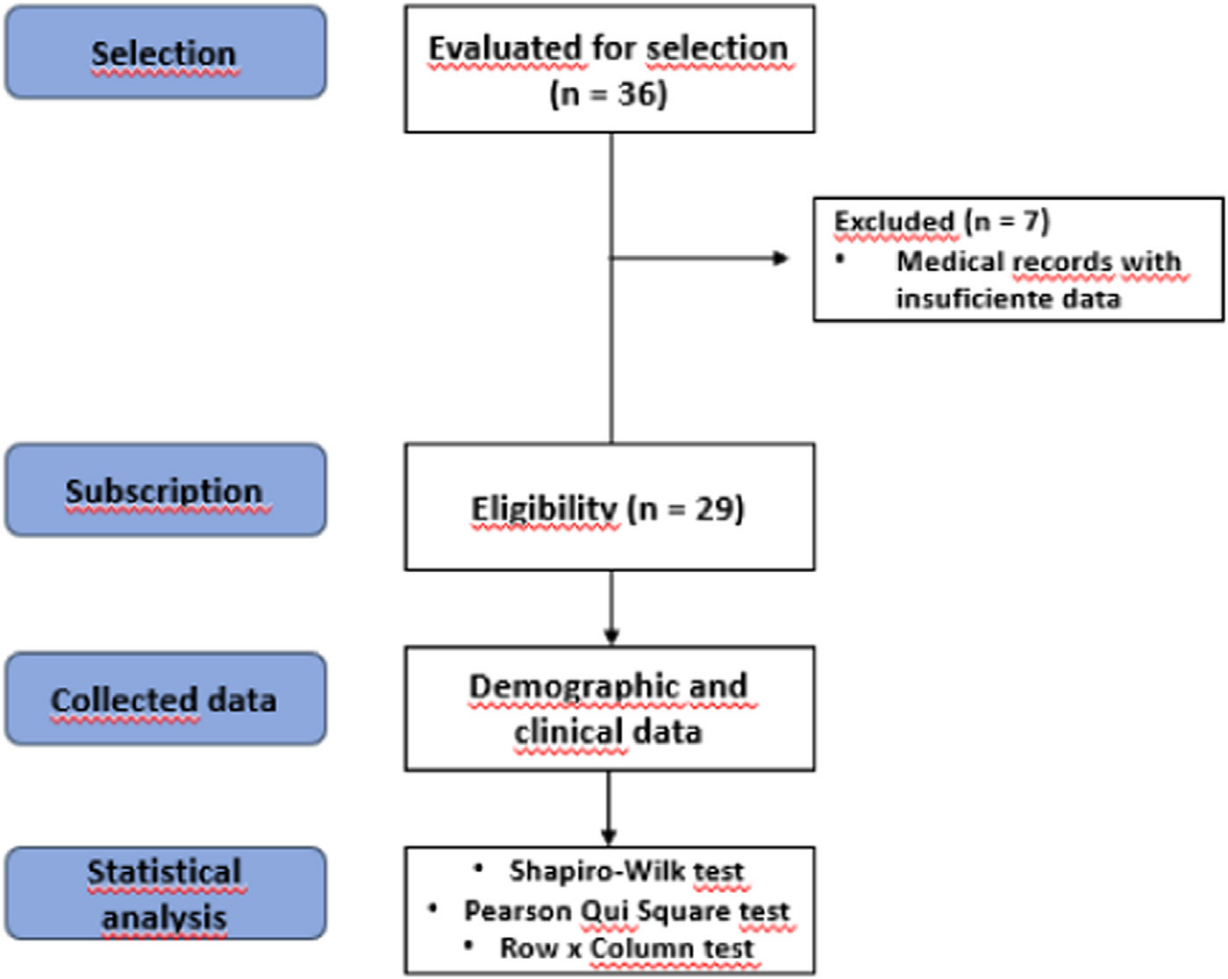
Study flowchart (CONSORT, 2010).

A total of 342 medical records of participants with CMF were selected, of which 55 used EF. Of these, 36 performed AT while using EF. Seven participants were excluded due to insufficient data in the medical records, resulting in a total of 29 participants (Fig. 1).

The sample consisted of 29 participants with a mean age of 12.1 ± 3.99 years and male predominance (16 medical records, 55 %). As for diagnosis, 11 (37 %) participants had hemimelia, 10 (35 %) had Congenital Femoral Deficiency (CFD) + hemimelia, 5 (17 %) had other diagnoses, and 3 (11 %) had CFD (Table 1).

**Table 1:** Sociodemographic and clinical characteristics.

		**Mean** ± **SD**	**Frequency n (%)**
**Age (years)**		12.1 ± 3.99	
**Time with EF (months)**		11.5 ± 4.46	
**AT time (months)**		6.8 ± 3.65	
**Gender**	Female		13 (45 %)
	Male		16 (55 %)
**Diagnostic**	CFD		3 (11 %)
	Hemimelia		11 (37 %)
	CFD+Hemimelias		10 (35 %)
	Other diagnostic		5 (17 %)
**Type of EF**	Circular		15 (51 %)
	Monolateral		14 (49 %)
**Localization of EF**	Femur		11 (38 %)
	Leg		8 (28 %)
	Femur+leg		5 (17 %)
	Leg+foot		5 (17 %)
**Purpose of surgery**	Bone lengthening		15 (52 %)
	Deformity correction		2 (6 %)
	Lengthening+correction		12 (42 %)
**Adverse events**	Infection		18 (62 %)
	Fracture-associated infection		3 (10 %)
	Pain		22 (76 %)
	ROM limitation		21 (72 %)
	Without complications		1 (3 %)
	Complications with surgical intervention		10 (37 %)
**Outcome of the rehabilitation**	Finished the process.		22 (76 %)
	Did not end due to clinical complications.		3 (11 %)
	Terminated for absences		4 (13 %)

n, Number of medical records; %, Percentage/frequency; SD, Standart Devitation; EF, External Fixator; AT, Aquatic Therapy; CFD, Congenital Femural Deficiency; ROM, Range of Motion.

As for the type of EF, 15 (51 %) participants used circular EF and 14 (49 %) used unilateral EF, with 11 (38 %) EFs in the femur, 8 (28 %) in the leg, 5 (17 %) in the femur + leg, and 5 (17 %) in the leg + foot.

The objective of the surgery in 15 (52 %) cases was bone lengthening, in 2 (6 %) it was deformity correction, and in 12 (42 %), lengthening + correction (Table 1).

The mean time of EF use was 11.5 ± 4.46 months, and the rehabilitation time in AT was 6.8 ± 3.65 months (Table 1).

During the rehabilitation process, skin pin-site infections occurred in 18 cases (62 %), fractures in 3 (10 %), pain in 22 (76 %), limited ROM in 21 (72 %), and only 1 (3 %) had no complications or adverse events. Of these, 10 cases (37 %) had complications requiring surgical intervention (Table 1).

Of the total, 22 (76 %) participants completed the rehabilitation process, and 4 (13 %) were removed from AT due to absences (not undergoing surgery).

The objectives established in the AT, in descending order of frequency, were: muscle activation, 29 (100 %) participants; weight bearing, 23 (79.3 %); mobility gain, 17 (58.6 %); balance, 16 (55.1 %); muscle stretching and gait with assistive devices, 15 (51.4 %); gait without assistive devices, 10 (34 %); stand, analgesia, and cardiorespiratory conditioning, 2 (6.80 %); and going up and down steps, 1 (3.4 %) (Table 2).

**Table 2:** AT purpose on rehabilitation.

**Objectives of the AT**	**Frequency n (%)**
Gait without assistive devices	10 (34 %)
Gait with assistive devices	15 (51.4 %)
Orthostatism	2 (6.80 %)
Weight bearing	23 (79.3 %)
Muscle stretching	15 (51.4 %)
Muscle activation	29 (100 %)
Mobility gain	17 (58.6 %)
Balance	16 (55.1 %)
Analgesia	2 (6.8 %)
Going up and down steps	1 (3.4 %)
Cardiorespiratory conditioning	2 (6.8 %)

n, Number of medical records; %, Percentage/Frequency; AT, Aquatic Therapy.

Variables, diagnosis, and EF type and location showed no association with the completion of the rehabilitation process, the need for surgical intervention, or the presence of complications such as fracture, pain, limited ROM, or infection. Complications and rehabilitation outcomes are not associated with diagnosis and EF type and location (Table 3).

**Table 3:** Association between diagnostic, EF type and localization with outcomes of AT.

	**Finished rehabilitation**	**Surgery intervention**	**Infection**	**ROM limitation**	**Pain**	**Fracture**
**Diagnostic**	p = 0.611	p = 0.213	p = 0.900	p = 0.651	p = 0.585	p = 0.949
**EF type**	p = 1.000	p = 0.450	p = 0.700	p = 0.682	p = 1.000	p = 0.626
**EF localization**	p = 0.448	p = 0.632	p = 0.931	p = 0.970	p = 0.710	p = 0.191

p < 0.05; EF, External Fixator; ROM, Range of Motion.

## Discussion

Few studies report the physiotherapy results in patients with CMF using EF, especially regarding AT. AADC has been pioneering this approach since 2011, with medical support and monitoring. Therefore, the objective of this study was to discuss the rehabilitation process of these patients, analyzing their main outcomes and complications in recent years at the institution.

There was a predominance of male participants with CMF, corroborating the latest epidemiological bulletin of the Ministry of Health,^10^ with an incidence of 24.43/10,000 live births, with a predominance of males (51.2 %) and 48.8 % female live births.

Regarding the diagnosis, most participants had tibial or fibular hemimelia and used a circular EF during the rehabilitation process. According to the literature, circular and unilateral EFs are widely used in patients with CMF;^4^ however, no data reinforce the main type of EF used, as it depends on the objective of the surgery.

As for EF location, the femur was the most frequent site and the main reason for surgery was bone lengthening. As for expected adverse events, there was a prevalence of infection, pain, and limited ROM. The main complications present during limb lengthening EF are not related to the surgical technique used, and the spectrum of potential complications is the same: muscle contracture, joint subluxation, axial deviation, nerve injury, vascular injury, premature union, delayed union, pin problems, fracture recurrence, joint stiffness, and pain.^11^ In the present study, the main complications were pin-site infection, fracture-associated infection, pain, and limited joint ROM.

Pain was the most recurrent complication in this study, found in 22 participants (76 %). It is the main cause of delayed functional acquisitions during the use of EF, especially circular,^12^ in addition to being cited as one of the main causes of discomfort.^13^ The literature demonstrates that aquatic physiotherapy can have positive effects on reducing pain in individuals with chronic dysfunctions, strengthening the rationale for using this therapeutic modality for this outcome.^14^ From a pathophysiological point of view, pain can be caused by increased nociceptive stimuli by interruption of painful sensitive afferent discriminative pathways (deafferentation pain) or by the association of both mechanisms.

No studies were found relating the risk of fractures or increased pain with AT in patients with EF. However, the temperature of the water has an analgesic effect, as it is a tactile stimulus that acts on the central nervous system through the gate control theory.^13^ Simply being in contact with the warm liquid medium, the patient may already experience a decreased pain perception. Turbulence is a great ally in pain treatment for providing several sensory stimuli that can be intensified with manipulation therapy to promote analgesia, being one of the main approaches at this stage of rehabilitation in this institution.^6^

ROM limitation was the second most recurrent complication in this study, due to the tensions exerted by the muscles in the regions to be lengthened or corrected.^11^ Therefore, the musculature has its heat conduction facilitated when immersed in heated water and the entire body warms, increasing blood flow. These factors, associated with the therapist's manipulation, increases the ROM.^15^

Pin-site infection is cited as the main complication, with a variable incidence that can affect up to 100 % of patients.^16^ Some variables affect its frequency, such as the duration of the process, materials used, type of surgical procedure, and pin region care. These data corroborate the findings of the present study, which reported pin-site infection as the third most frequent complication. In some cases, infections were associated with fractures or required surgical intervention. The onset of pin-site secretions is decisive for the suspension of treatments in the liquid medium, requiring medical clearance to return to therapy.

The literature reports fractures as the main complication in limb correction and lengthening in children.^17^ However, no correlations were found between lengthening, diagnosis, and fractures, which reinforces the variable onset of complications. Congenital diseases correlate to a more fragile bone, which can predispose to fractures during the lengthening process.

Rehabilitation during bone lengthening or correction can be divided into four phases: 1) Hospitalization (post-operative days 1–3), 2) Lengthening or correction, 3) Consolidation, and 4) Post-device removal.^2^ During phases 2 and 3, the patients will have the device attached to the limb. Physiotherapy aims at improving ROM, decreasing pain and atherogenic muscle inhibition, preventing muscle trophic changes, and facilitating bone consolidation through the piezoelectric effect promoted by weight bearing and muscle traction.^2^ In phase 2, the main objectives are to maximize joint mobility and maintain muscle tone and flexibility, and in phase 3, the objective of physical therapy is to maximize ROM and improve muscle strength.^2^ Some authors reinforce the importance of AT in helping bone consolidation and early weight bearing to optimize the rehabilitation process.^3^

The main objectives established in the AT rehabilitation process in this study were muscle activation, weight-bearing, mobility gain, balance, muscle stretching and gait with assistive devices, gait without assistive devices, standing, analgesia, cardiorespiratory conditioning, and going up and down steps. The most frequent objectives were muscle activation, weight-bearing, and mobility gain, which play a crucial role in helping bone consolidation and ROM maintenance and are related to the main complications and limitations present in the rehabilitation process of these patients.

The physical and thermodynamic effects exerted by heated water on the immersed body can facilitate or hinder movements depending on the depth and proposed activity and may accelerate the rehabilitation process of these individuals, bringing benefits such as improved ROM, muscle activation, balance, and gait performance.^18^

Hydrostatic pressure is stated in Pascal's principle as: “Pressure applied to a fluid in a closed container is transmitted equally to every point of the fluid and to the walls of the container”. The greater the depth, the greater the pressure exerted on the body, thus improving venous return, and reducing edema. It also acts as a resistance to movement, which associated with the action of viscosity, promotes greater neuromuscular activation than on the ground, where the patient has greater difficulties due to pain and arthrogenic inhibition.^7^

**Fig. 2:**
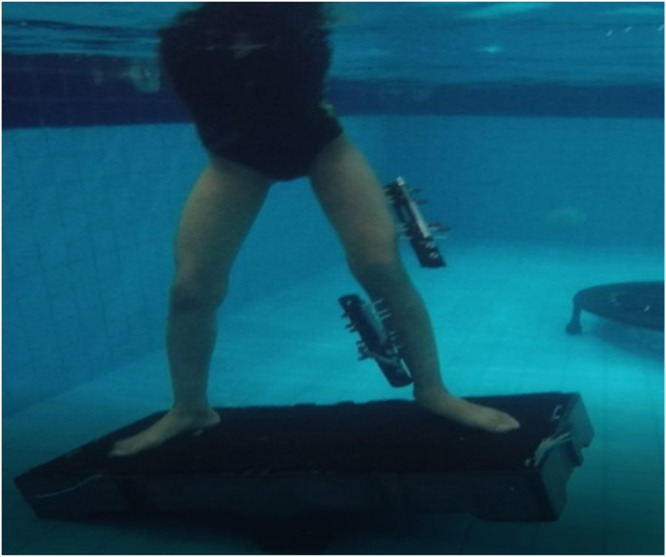
Balance training and weight bearing using the physical properties of water.

Buoyance compensates for the EF weight, improves muscle activation, and allows weight bearing (Fig. 2) at the beginning of the limb lengthening and/or correction process even in case of weight-bearing restrictions on the ground. With water up to the nipple line, which equates to 35 % weight bearing, patients can use water buoyancy to protect their tissues against gravity, decrease compressive forces for ambulation, and improve cardiovascular conditioning.^2^

Hydrodynamic properties optimized the rehabilitation process of these patients, with 76 % of them completing the rehabilitation process for reaching the established objectives, 13 % not concluding for not adhering to the treatment, with an excessive number of absences, and only (11 %) not completing the rehabilitation process due to clinical complications.

The current study demonstrated that it is possible to treat this population with aquatic physiotherapy. In this way, the authors can aim for new research to verify whether this treatment can really accelerate the rehabilitation process and measure the results of the exercises. This will benefit the patient and may reduce costs and optimize the flow of the public health system.

## Conclusions

The current study was able to describe the rehabilitation process of people with CMF during the use of EF in AT. There was no association between EF type and location and diagnosis and the need for surgical interventions and adverse effects during AT.

## Authors' contributions

Caio Roberto Aparecido de Paschoal Castro: Conceptualization; Project administration; Software; Writing Original and Review & Editing; Methodology; Investigation.

Alessandra Mitie Kakihata: Conceptualization; Writing Original; Investigation.

Carla Borges Fleuri de Barros: Conceptualization; Writing Original; Investigation.

Monica Gonçalves: Conceptualization; Writing Original; Investigation.

Beatriz Carvalho de Souza: Writing Original.

Larissa Soares da Silva: Writing Original.

Douglas Martins Braga: Conceptualization; Supervisor; Writing Original.

## Declaration of competing interest

The authors declare no conflicts of interest.

## Appendix Supplementary materials

Image, application 1

## References

[1] Rodrigues LS, Lima RHS, Costa LC, Batista RFL. (2014). Características das crianças nascidas com malformações congênitas no município de São Luís, Maranhão, 2002-2011. Epidemiol. Serv Saúde Brasília..

[2] Bhave A, Baker E, Campbell M. (2016). Physical therapy during limb lengthening and deformity correction: principles and techniques. Pediatric Lower Limb Deformit.

[3] Pereira EC, Candeloro JM. (2005).

[4] Fernandes AC, Ramos ARC, Filho MCM, Jesus M (2014). Reabilitação (Rehabilitation). Manole. Ed.

[5] Hasler CC, Krieg AH. (2012). Current concepts of leg lengthening. J Child Orthop.

[6] Becker EB. (2009). Aquatic therapy: scientific foundations and clinical rehabilitation applications. P M R.

[7] Duarte M. (2004). http://www.eefe.usp.br/.

[8] Caromano FA, Nowotony JP. (2002). Princípios físicos que fundamentam a hidroterapia. (Physical principles underlying hydrotherapy). Fisioterapia Brasil.

[9] Goldman V, Weiss PL, Weil Y, Eylon S. (2023). Hydrotherapy for patients with external fixation: effect on infectious events. J Pediatr Orthop.

[10] Ministério Da Saúde. Anomalias congênitas no Brasil, 2010 a 2019: análise de um grupo prioritário para a vigilância ao nascimento. (MINISTRY OF HEALTH. Congenital anomalies in Brazil, 2010 to 2019: analysis of a priority group for birth surveillance). Brasília, 2021. 22 pages. Available at: https://www.gov.br/saude/pt-br/media/pdf/2021/marco/3/boletim_epidemiologico_svs_6_anomalias.pdf, Accessed on Nov 04, 2021.

[11] Paley D. (1990). Problems, obstacles, and complications of limb lengthening by the Ilizarov technique. Clin Orthop Relat Res.

[12] Imamura M, Targa WHC, Teixeira MJ, Yeng LT, Imamura ST. (1995). Dor e fixadores externos: avaliação e tratamento (Pain and external fixators: assessment and treatment). Acta Fisiátrica.

[13] Júnior EAS, Campos PHS, Mourão RLPT, Baumfeld DS, Campos TVO, Andrade MAP. (2018). Considerações sobre fixadores externos sob a perspectiva do paciente (Considerations on external fixators from the perspective of the patient). Arch Health Investigation.

[14] Antunes M, Vertuan M, Miquilin A, Pasqual Marques A, Morales R (2021). Effect of aquatic physiotherapy on pain and quality of life in elderly women with fibromyalgia. Ann Rheum.

[15] Cunha MG, Caromano FA. (2003). Efeitos Fisiológicos da Imersão e Sua Relação Com a Privação Sensorial e o Relaxamento Em Hidroterapia (Physiological effects of immersion and its relationship with sensory deprivation and relaxation in hydrotherapy). Revista de Terapia Ocupacional Da Universidade de São Paulo. J Occupat Therapy of the University of São Paulo.

[16] Pin T, Dyke P, Chan M. (2006). The effectiveness of passive stretching in children with cerebral palsy. Dev Med Child Neurol.

[17] Hosny GA. (2020). Limb lenghtening history, evolution, complication, and current concepts. J Orthop Traumatol.

[18] Calder P, Faimali M, Goodier WD. (2019). The role of external fixation in paediatric limb lengthening and deformity correction. Injury.

