# Supplementary material for: Aquatic therapy in congenital malformation during the use of external fixator for bone lengthening: It is possible?
Source: Clinics (Sao Paulo). 2024 Jun 18;79:100416. doi: 10.1016/j.clinsp.2024.100416 (PMC11237682; doi:10.1016/j.clinsp.2024.100416)
Supplement: Supplementary file 1 [file mmc1.pdf]

November 17th, 2023

Caio Roberto Aparecido de Paschoal Castro
Avenida Professor Ascendino Reis, 724
São Paulo - SP
caio.paschoal11@hotmail.com
+55 11 960466070
17/11/2023

Julia Maria D'Andrea Greve, PhD
Clinics Journal
Hospital das Clínicas da Faculdade de Medicina de São Paulo
São Paulo - SP

Subject: Request to Waive Article Processing Fees

Dear Dra. Julia Maria D'Andrea Greve

On behalf of the authors I am pleased to submit our manuscript entitled: ***"Aquatic therapy in congenital malformation during the use of external fixator for bone lengthening: it is possible?"*** for consideration for publication in *Clinics Journal.*

Children with congenital malformations often undergo bone correction surgeries throughout their lives, due to musculoskeletal changes. One of the alternatives for bone corrections is the use of an external fixator.
When using this device, there is a risk that these children may experience pain, decreased range of motion, infections, and fractures.
After surgery, they need to go through a rehabilitation process to improve functionality and reduce the risks to which they are exposed. In the literature there are rare exceptions that aquatic physiotherapy is a treatment option for these children at this time, however it is known that this therapeutic modality can bring several benefits to this population.
The institution that proposes the current study is a **pioneer** in Brazil in using aquatic physiotherapy as a treatment for children with congenital malformations during the use of external fixators for bone corrections. Therefore, the objective of this study is to demonstrate the potential of aquatic physiotherapy for this population, so that other rehabilitation centers can benefit more children with the same dysfunction.

We are submitting this work for publication in your journal and would like to request the waiver or discount on the article processing fees associated with its publication.
We understand that article processing fees play a crucial role in supporting the operations and maintenance of publications like yours. However, as scientists from a middle-income country (Brazil) who work at a non-profit institution, in a project with no grants or research funding associated, we face financial constraints that make it challenging to cover such expenses. Consequently, I am kindly requesting your consideration in waiving the article processing fees for our submission.

vida é movimento

We firmly believe that our article holds significant relevance and value to the readers of Clinics Journal. By publishing this article in your journal, we are confident it will contribute positively to the ongoing discussion on rehabilitation of these children.
Given our conviction in the benefits of open science to our society and the desire to share our reserch with a wider audience, we sincerely hope that you will consider our request. We are committed to providing any additional information or documentation that may be required to support our appeal.

Thank you for taking the time to consider our application. We look forward to a positive response and the opportunity to contribute to the continued success of Clinics Journal, keeping the high standards you uphold and providing quality content to readers.

Yours sincerely,

Caio Roberto Aparecido de Paschoal Castro, MSc, PT.
